# Glenoid Component Position Does Not Affect Short-Term Clinical and Radiologic Outcomes in Total Shoulder Arthroplasty

**DOI:** 10.3390/jcm10245773

**Published:** 2021-12-09

**Authors:** Maciej J. K. Simon, Helen Crofts, Treny Sasyniuk, Kayla Johnston, Derek Plausinis, Zane D. S. Zarzour, Fay Leung, Patrick Y. K. Chin, William D. Regan

**Affiliations:** 1 Department of Orthopaedics, Chan Gunn Pavilion, University of British Columbia, 2553 Wesbrook Mall, Vancouver, BC V6T1Z3, Canada; 2 Department of Orthopaedics and Trauma Surgery, University Medical Center Schleswig-Holstein-Campus Kiel, Arnold-Heller-Strasse 3, 24105 Kiel, Germany; 3 Rebalance MD, 3551 Blanshard St. #104, Victoria, BC V8Z 0B9, Canada

**Keywords:** total shoulder arthroplasty, glenoid position, clinical outcomes, radiological outcomes

## Abstract

Background: Malpositioning of the glenoid component in total shoulder arthroplasty (TSA) remains the primary source of loosening. The purpose of this study is firstly, to quantify postoperative glenoid component position in patients having a TSA and secondly, to explore whether glenoid component radiolucency is associated with glenoid position, clinical outcomes and patient-reported measures in the short-term (two year) follow-up period. Methods: This study was a sub-study of a larger clinical trial that included patients who underwent a TSA and who were randomized into two different glenoid types with a minimum two-year follow-up period. Post-operative radiographic assessments (six weeks and two years) were used to measure glenoid component position (version, inclination, offset) and humeral head centering anterior–posterior (AP) and superior–inferior (SI), and to assess glenoid component radiolucent scoring (modified Lazarus). Pre-operative X-rays were used to measure glenoid version, inclination and Walch classification. Patient-reported measures (PROMs) included the EQ-5D health slider and the Western Ontario Osteoarthritis (WOOS) and American Shoulder and Elbow Surgeons (ASES) score and were captured at baseline and two years postoperative. Clinical outcomes including range of motion and complications were also documented. Statistical analysis included t-tests and regression modeling. Results: Ninety-one patients with an average age of 69.9 ± 6.2 years were included in this study. Glenoid component position improved significantly in version (−19.4 ± 8.6° to −17.7 ± 8.5°; *p* < 0.045) and inclination (11.5 ± 7.1° to 5.9 ± 6.3°; *p* < 0.00001) from preoperative to six weeks postoperative. Glenoid component offset in SI and humeral head centering in AP remained unchanged throughout the follow-up. Radiolucency (Lazarus classification) was recorded in 21 cases (17.3%) with a Lazarus score of 1 (15 cases) and 2 (6 cases). The EQ-5D health slider, WOOS and ASES, and ROM confirmed continuous improvements from the preoperative scores to the two-year follow-up (*p* < 0.05). Regression models showed no correlation between glenoid component radiolucency at two years and the postoperative week six glenoid component position; however, female gender was a significant variable. Conclusion: Glenoid component changes from its original native glenoid were observed following TSA. Glenoid inclination was improved more than version from baseline, and the humeral head remained well-centered in AP and SI at two years. Radiolucency of the glenoid at two years is not negatively associated with PROMs or component position; however, female gender was identified as a significant predictor and warrants further investigation. Complications are not associated with glenoid position or radiolucency, but longer-term follow-up is required.

## 1. Introduction

Glenoid version, inclination and wear patterns are important factors to consider when performing an anatomic total shoulder arthroplasty (TSA) [1,2,3]. The Walch classification outlining the native glenoid morphology in glenohumeral arthritis is commonly used [4]. A modified Walch classification system proposed by Bercik et al. added additional glenoid wear types [5]. Significant glenoid retroversion, according to Walch, is correlated with increased glenoid component loosening [6] and biconcave glenoids are associated with a high revision rate [7].

Glenoid component malposition is a contributing factor to loosening and poorer clinical outcomes [1,6]. Gregory et al. confirmed that an inferiorly inclined glenoid component was associated with increased radiolucent lines [1]. Other research has shown no association between glenoid component version and clinical outcome scores [8,9]. For these reasons, it is important to examine and quantify the glenoid component position over time, and further, to determine whether glenoid component position impacts clinical or patient-reported outcomes.

The purpose of this study is firstly, to quantify glenoid component position in patients having a TSA and secondly, to explore whether glenoid component radiolucency is associated with glenoid position, clinical outcomes or patient-reported measures in the short-term (two year) follow-up period. We hypothesize that changes will be observed in glenoid component position and that glenoid component radiolucency is associated with glenoid position or PROMs at two years postoperative.

## 2. Materials and Methods

### 2.1. Patients

Patients included in the current study were part of a prospective, randomized controlled multi-center study comparing two glenoid components [10]. This study was a retrospective analysis of prospectively collected data. Ethics approval for the trial was granted by the University of British Columbia Clinical Research Ethics Board and the study was registered with clinicaltrials.gov (Date: 27 February 2012) (NCT01539122). Informed consent for participation was obtained from all participants. The study was carried in accordance with the latest Declaration of Helsinki.

Patients presenting to one of five shoulder surgeons between June 2012 and December 2016 were screened for eligibility [10]. Inclusion criteria were age (19–79) and primary glenohumeral osteoarthritis (GH-OA). Exclusion criteria were patients with significant glenoid bone loss on a pre-operative CT scan or intra-operatively, major joint trauma, avascular necrosis, rotator cuff or inflammatory arthropathy, chronic dislocations, massive cuff tear, previous shoulder surgery (other than arthroscopic debridement or acromioplasty), active joint or systemic infection, muscle paralysis, Charcot arthropathy, life expectancy <2 years or who were unable to read or speak English [10].

**Figure 1:**
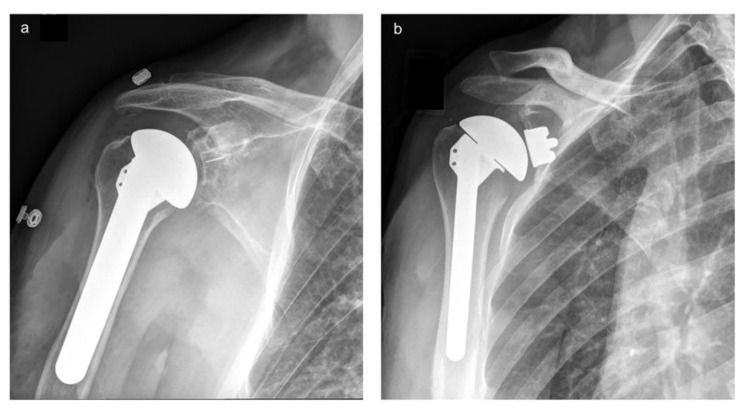
Radiographic images demonstrate an anatomic total shoulder arthroplasty of a right shoulder with part (**a**) a cemented polyethylene (PE) and part (**b**) a trabecular metal (TM) glenoid.

The glenoid bone stock was assessed intraoperatively prior to randomization. Patients with a contained defect suitable for an anatomic keeled implant were deemed to have adequate bone stock and were randomized to receive one of two glenoid implants. The two glenoid groups were an uncemented second-generation Trabecular Metal^®^ (TM) glenoid (Zimmer Biomet; Warsaw, IN, USA) and a cemented all-polyethylene (PE) glenoid (Zimmer Biomet; Warsaw, IN, USA) (Figure 1). Randomization was performed using a central data center (EmPOWER Health Research; London, ON, Canada) which was responsible for generating the randomization codes, which were stratified by the surgeon. All patients received an uncemented Bigliani-Flatow humeral prosthesis (Zimmer Biomet; Warsaw, IN, USA) [10].

Surgical techniques and postoperative details are described in Chin et al. [10]. In the literature, glenoid version averages about 1.23° retroversion with a range from −9.5 to 10.5° [11] and average inclination angles are approximately 1.3° (range from −3 to 7°) [12]. We aimed to correct the glenoids to neutral, but a level of <10° was accepted to preserve anterior bone stock. Glenoid component inclination was aimed for 5°.

Shoulder X-rays were taken in true–anterior posterior (AP), lateral and axial views at baseline, six weeks and two years postoperative. Postoperative day one images were not used in this study because they were only available in AP and not in axial views as patients’ pain levels did not allow for second plane imaging.

### 2.2. Radiographic Measures

**Figure 2:**
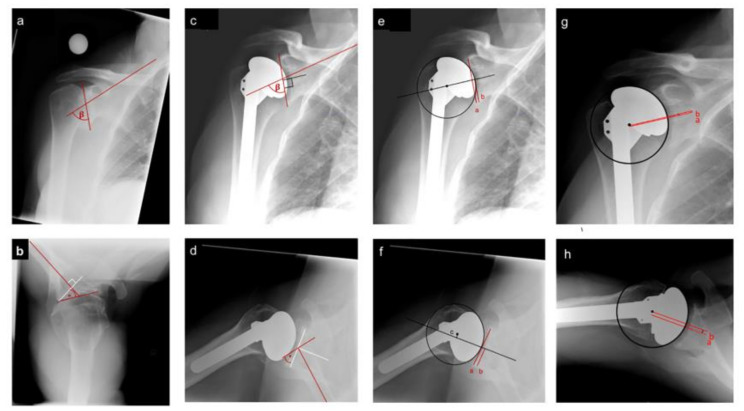
Radiographic measurements: (**a**) Pre-operative inclination = 90−β, where β is the angle between the supraspinatus fossa and a line from the superior to inferior glenoid. (**b**) Pre-operative version = the angle (*) between a line connecting the anterior to posterior glenoid and a perpendicular line to the mid-scapular blade. (**c**) Glenoid component inclination = 90−β. Angle β is the angle between the supraspinatus fossa and a perpendicular line to the mid-component axis. (**d**) Glenoid component version = the angle (*) between a line perpendicular to the mid-component axis and a perpendicular line to the mid-scapular blade. (**e**) Glenoid component offset SI = difference between the midpoint of a line connecting the superior to inferior glenoid and length of a line connecting the midpoint of the glenoid component to the inferior glenoid. (**f**) Glenoid component offset AP = difference between the midpoint of a line connecting the anterior to posterior glenoid and length of a line connecting the midpoint of the glenoid component to the posterior glenoid. (**g**) Humeral component offset SI = the difference between line A (a line through the center of the glenoid component) and line B (a parallel line to line A, from the center of the humeral component). (**h**) Humeral component offset AP = the difference between line A (a line through the center of the glenoid component) and line B (a parallel line to line A, from the center of the humeral component).

The primary outcome is glenoid component position. Glenoid position was evaluated radiographically using the following measurements: glenoid component version, inclination [13,14] and offset (glenoid component and humeral head) [15] (Figure 2) [4,5,13,14]. Measurement techniques are outlined in Figure 2. Glenoid retroversion was scored as negative and anteversion recorded as a positive value. Secondary radiographic measurements included Walch classification [4] for native glenoid wear, which was evaluated based on the pre-operative images, and glenoid component radiolucency, which was evaluated according to the Lazarus classification [16]. Assessment of Trabecular Metal^®^ (TM) glenoid component loosening was, by necessity, modified from the Lazarus score to include osteolysis around the (TM) component (criteria listed in Table 1 legend).

Two raters (MS and HC) independent from the main trial completed all radiographic measurements. The independent raters were blinded to the patient clinical history and scores. Radiographic measures were standardized and reviewed by both raters prior to evaluation. One rater was an orthopaedic fellow and the other was an orthopaedic resident. Each observer/rater was blinded to the other’s measurements.

### 2.3. Clinical Outcomes and PROMs

**Figure 3:**
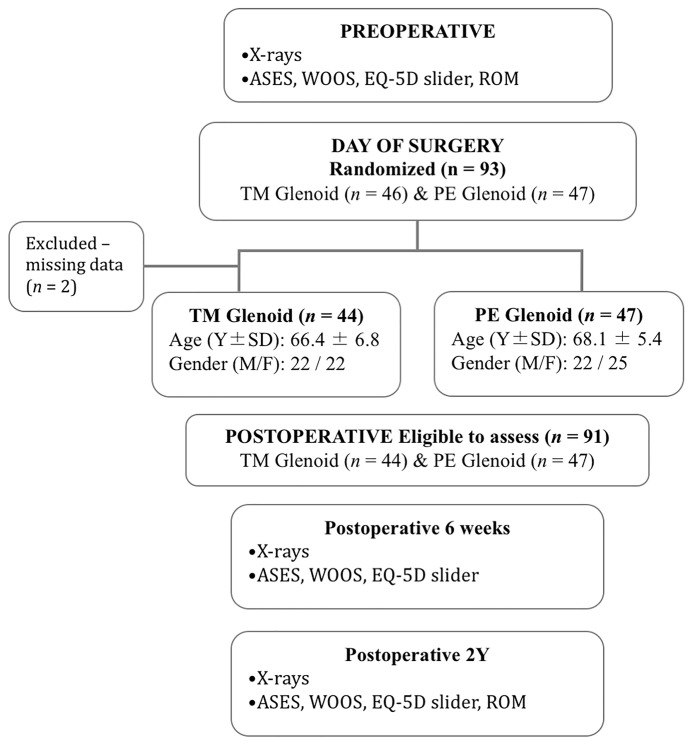
Consort Flow Diagram. American Shoulder and Elbow Surgeon (ASES) score [17]; Western Ontario Osteoarthritis of the Shoulder (WOOS) Index [18], quality of life scores, and EQ-5D health slider (0 = worst imaginable health status and 100 = best imaginable health status) [19].

Demographic variables included age, gender, body mass index (BMI), dominant and operated arm. Information related to the implants and surgical procedures were documented on the day of surgery. Clinical assessment and patient-reported outcome measures (PROMs) were performed pre-operatively (baseline) and postoperatively at six weeks and then at two years (Figure 3). The assessments included range of motion (ROM) with a goniometer, the American Shoulder and Elbow Surgeon score (ASES) [17], the Western Ontario Osteoarthritis of the Shoulder (WOOS) Index [18], and the EQ-5D health slider (0 = worst imaginable health status and 100 = best imaginable health status) [19]. Complications and reoperations were also captured.

### 2.4. Statistical Analysis

Descriptive statistics were calculated for demographic, radiological and clinical variables, as well as change from pre-operation to two-year post-operation for all clinical and patient-reported outcomes. Changes in glenoid position variables between pre-operative and six weeks postoperative, and between six weeks and two years postoperative were analyzed via t-test to assess the significance of change at each time point. Within-group comparisons for ROM and PROMS between baseline and two years postoperative were made via paired t-tests. Inter-rater reliability of radiographic evaluations was assessed via intraclass correlation coefficient (ICC).

Glenoid component radiolucency (binary) was regressed on 6-week glenoid version, glenoid inclination, and humeral head centering (AP and SI views in one model), using logistic regression controlling for age, sex, BMI and dominant hand operated (yes/no) and was also adjusted for deltas (pre-to-postop) and pre-operative Walch score. The continuous PROMS measures such as WOOS were regressed on two-year glenoid component radiolucency in linear regression models adjusted for age, sex, BMI and dominant hand operated (yes/no). Logistic model fit was assessed via the Hosmer and Lemeshow goodness of fit test [20]. Linear model fit was assessed via normal quantile–quantile plots of the standardized residuals.

Analyses were performed on the entire study population, as well as stratified by glenoid type: PE vs. TM. Analyses were performed using SAS version 9.4 (SAS Institute Inc., Cary, NC, USA).

## 3. Results

### 3.1. Patients

Ninety-one patients were included in this study, 44 with a TM-glenoid and 47 with a PE-glenoid (Figure 3). Two cases (TM-glenoid) were excluded because radiographic data were unavailable at the time this study was conducted. The average age of all patients was 67.3 ± 6.2 years. Forty-seven (51.6%) patients were male and 44 (48.4%) were female. BMI average was 28.6 ± 5.6%. In 40 (45.5%) of cases, the operative arm was the patient’s dominant arm. No significant differences were noted for age, gender, BMI or operative side between the two glenoid groups.

### 3.2. Radiologic Outcomes

Preoperative imaging demonstrated an average glenoid version of −19.4 ± 8.6° and glenoid inclination of 11.5 ± 7.1° (Table 1). Glenoid wear classification according to Walch [4] demonstrated the following glenoid types: A1 = 29, A2 = 6, B1 = 19, B2 = 30, B3 = 1, C = 5, D = 0. One case had preoperative radiographs missing.

Postoperative week six measures demonstrated that the glenoid component was positioned at −17.72 ± 8.53° version and an inclination of 5.87 ± 6.31° which was significantly different than the pre-operative measures (*p* < 0.045 and *p* < 0.00001, respectively) (Table 1). Overall humeral head centering demonstrated minimal displacement according to measurements of the position in the anterior–posterior (AP) and superior–inferior (SI) views (Table 2). One case demonstrated radiolucency around one peg (Lazarus 1) (Table 1). There were no clinically significant differences identified between glenoid types.

Postoperative year two measurements showed no significant change from six weeks postoperative in the glenoid component version (−18.24 ± 9.00°); however, inclination (6.38 ± 6.33°) was significantly different (*p* < 0.0001) (Table 1). The humeral head remained well centered in AP (1.52 ± 2.91mm) and SI (1.38 ± 3.03mm) positions at the two-year follow-up (Table 2). However, significant differences were detected in glenoid component offset in AP and humeral head centering on SI. Radiolucency was observed in 21 of 68 (30.9%) cases, Lazarus 1 (*n* = 15) and Lazarus 2 (*n* = 6) at the two-year mark (Table 1). There were more radiolucencies in the PE glenoids (*n* = 13) versus TM glenoids (*n* = 8), but no differences between glenoid component type and component position were detected.

Except for the six-week postoperative AP humeral head centering (0.277), all measurements demonstrated moderate (0.5–0.75) or good (>0.75–0.9) inter-rater reliability (Table 3).

### 3.3. Clinical Outcomes

PROMs demonstrated a significant improvement from pre-operative status overall and in subgroup analyses. Combined ASES and WOOS scores significantly improved from 35.47 ± 18.04 and 29.07 ± 18.37 preoperatively to 89.07 ± 14.07 and 92.40 ± 11.95 at the two-year follow-up, respectively (*p* < 0.0001 and *p* < 0.0001). Significant improvements were also observed with the EQ-5D health slider score from baseline to two-year follow-up (72.4 ±16.5 to 81.3 ±12.8; *p* < 0.001). There were no statistically or clinically relevant differences in PROMs between the two glenoid types at two-year follow-up [10].

Forward elevation (FE) improved significantly from 102.0 ± 27.17° to 139.00 ± 18.83° (*p* < 0.0001) in the two-year follow-up. External rotation (ER) also significantly increased from 16.56 ± 18.64° preoperatively to 51.06 ± 18.52° at two years postoperative (*p* < 0.0001). There were no statistically or clinically relevant differences in PROMs between the two glenoid types at two-year follow-up [10].

### 3.4. Complications

There were in total 11 cases with a complication (TM = 5 and PE = 6) [10]. Six cases required a reoperation—none related to the glenoid component. The TM group had two periprosthetic fractures (one treated operatively), two cases with aseptic humeral stem loosening which were exchanged to a cemented humeral stem, and one case with dislocation (no rotator cuff damage, just closed reduction). The PE group had three cases with a subscapularis tear within the first three postoperative months (two traumatic due to a fall, one atraumatic). These had to be revised. The other three complications were caused by minor periprosthetic fractures and were treated conservatively.

### 3.5. Regression Analyses

Logistic regression models showed no correlation between glenoid component radiolucency at two years and post-operative week six glenoid component position when controlled for preoperative glenoid wear, age, BMI and whether the operated arm was the dominant arm (Table 4). Interestingly, female gender was identified as having a statistically significant association with glenoid radiolucency at two years across all models (Table 4).

Linear regression models for the two-year PROMs showed no correlation between glenoid component radiolucency at two years when controlled for gender, age, BMI and whether the operated arm was the dominant arm (Table 5).

## 4. Discussion

The current study quantified changes in glenoid component position compared with native glenoid following TSA. Glenoid component version (*p* = 0.045) and inclination (*p* < 0.00001) were changed significantly with the latter demonstrating greater post-operative correction at six weeks. The humeral head remained well centered in AP and SI at two years. There was no association between glenoid radiolucency and component position or PROMs; however, female gender was identified as a significant predictor. ROM and PROMs improved over the study period and were unaffected by glenoid component position or type. Complications were not related to glenoid position.

Glenoid exposure during TSA can be challenging and may influence optimal intra-operative positioning of the glenoid component, which is paramount for component longevity [21,22]. The current study results demonstrate that the average glenoid component version improved marginally, whereas glenoid component inclination was closer to the neutral position. The reason for this is multifactorial. First, intraoperative glenoid exposure can be difficult to achieve due to the surrounding soft-tissue bulk and hence the preparation and implantation of the component may be compromised [21,22]. Second, excessive reaming of the glenoid, particularly anteriorly, is avoided as this can lead to loss of bone stock and more cancellous bone exposure, which results in reduced stability than cortical bone, potentially causing decreased glenoid component fixation [3]. Another reason for misinterpretation of the angles can be due to intraoperative patient positioning, particularly as positioning in a “lazy beach chair” or a beach chair position can vary significantly from patient to patient depending on their body habitus. Finally, postoperative imaging can be difficult to interpret as X-ray projections and implants are not standardized, causing measuring errors specifically for glenoid version [14]. Postoperative CT scans could reduce projection and measuring errors on X-rays, but CT scans are not always accessible, are expensive, and patient safety remains an issue [23].

The modest but statistically significant correction of glenoid component version had no effect on clinical outcome measurements, including ROM and PROMs, regardless of glenoid component type. This contrasts with the study by Gregory et al. who established a correlation between poorly positioned glenoid components with clinical and radiological outcomes [1]. However, they also reported a very wide range of poor glenoid component version from 17° anteversion to 32° retroversion and concluded that even experienced surgeons have difficulty positioning the glenoid component in a neutral position. In the current study, there were a total of seven patients with glenoid component retroversion >30° which supports the theory that two-year results are not adversely affected with glenoid implants positioned at 30° of retroversion. Preoperative retroversion and posterior glenoid wear can make glenoid component placement and neutralization of retroversion difficult when utilizing standard glenoid components. Posteriorly augmented glenoid components have been developed and are being used, particularly with extremely retroverted glenoids (Walch B2 and B3). The use of posterior augments improving version correction has been successfully demonstrated by Ricchetti et al. [24]. However, long-term studies analyzing the clinical outcomes are not yet available. Similar improvements were identified by Ho et al. with the use of posterior augmented glenoids for correction of retroversion and humeral head subluxation [25]. These implants are more expensive, and their use may be called into question by this study. The results of our study confirm that even with modest but significant correction of glenoid version, PROMs improved significantly post-operatively and not at the expense of increased component radiolucency, nor instability.

Another factor contributing to glenoid component loosening is rotator cuff integrity. Postoperatively, subscapularis tendons may fail to heal or can tear despite a good repair. In addition, the supraspinatus tendon can tear due to increased glenohumeral component friction due to overstuffing the joint [26], or poorly tensioned soft tissues [27]. Apart from the one atraumatic and two traumatic rotator cuff tears, the current two-year follow-up data do not demonstrate any further rotator cuff tears clinically nor radiographically. Humeral head centering measurements demonstrate stable positioning in the superior–inferior and anterior–posterior assessment with only marginal changes from six weeks postoperative to two-year follow-up images. Proper humeral head centering is reflected indirectly via good clinical outcomes and continuous improvements in ROM or PROMs [28]. The current data could not identify any negative correlation between glenoid component position and clinical outcomes. These findings contradict other studies that report poorer clinical outcomes with inferior glenoid version correction [1] or link better clinical outcomes to correction of glenoid retroversion and humeral head subluxation [25]. Further and longer follow-up studies on humeral head centering should explore the couple forces of the rotator cuff potentially contributing to radiolucencies around the glenoid component.

Radiolucency around the posterior glenoid component is important to detect and quantify as it can identify early problems with loosening. The original radiolucency classification described by Lazarus et al. [16], was used in the current study for the cemented PE glenoids and an adapted version was created for the TM glenoids. There were 21 cases with a minor radiolucency (Lazarus 1 = 15 and Lazarus 2 = 6) around the glenoid components. No clinically significant difference was observed between PE (*n* = 13) and second-generation TM glenoid components (*n* = 8). Second generation TM glenoids, with the addition of a porous tantalum keel, has improved the component design of the first generation where early loosening and high rates of revision surgery were reported [29,30]. However, these studies reported longer follow-up times and thus, the radiolucency and survival of the current TM glenoids may deteriorate with longer follow-up.

One interesting finding with respect to glenoid component radiolucency, is that female gender had a statistically higher odds ratio in the six-week regression models for component position (glenoid version, inclusion and humeral head centering). Reasons to explain this finding are unclear and warrant further investigation, notably longer-term follow-up.

The current study has limitations. The radiographic images were obtained in multiple facilities as it was a multi-center study and strict standardization of shoulder X-ray protocol was not feasible. Furthermore, non-standardized pre- and post-operative radiographs might have a bearing on the marginal improvement between pre-operative and postoperative changes in the glenoid version. CT scans are the gold standard to identify changes in bone and particularly radiolucent lines around glenoid components [23]. However, CT scans were not used in this analysis, firstly because only pre-operative CT scans were obtained and available, but we wanted to have a comparison in the same radiological modality from preoperative to the final follow-up visit. Secondly, CT scans can be difficult to obtain, particularly for rural living patients and the increased expenses that entail [31]. Thirdly, CT scans have an increased radiation exposure. Nonetheless, the inter-rater correlation coefficient demonstrated good correlations for the assessment of glenoid version and inclination scores, reducing this major bias. Furthermore, the follow-up of two years is short and the likelihood of finding increased radiolucencies of the glenoid component increases with time. Therefore, a correlation between the glenoid position at implantation and loosening might be not detected at the current time point. Finally, assessment of radiolucency around a second-generation TM glenoid is not standardized and is complicated due to the radiodense material. As a result, an adaption of the Lazarus classification [16] was created to accommodate for the tantalum metal block in order to assess loosening in this subgroup.

The strengths of the presented study include the collection of prospective data within a level I study. Results indicate good ICC for radiographic measures. Patients were closely followed with multiple PROMs and ROM, and there was minimal patient drop out.

## 5. Conclusions

Changes in glenoid component position compared with the preoperative native glenoid were observed following TSA. Glenoid inclination was improved more than version, and the humeral head remained well-centered in AP and SI at two years. Radiolucency of the glenoid at two years is not negatively associated with PROMs or component position; however, female gender was identified as a significant predictor and warrants further investigation. Complications were not associated with glenoid position or radiolucency, but longer-term follow-up is required.

## Figures and Tables

**Table 1:** Preoperative glenoid wear (Walch classification) assessment of the native glenoid and glenoid component radiolucency (Lazarus classification [16]) at 6-week and 2-year follow-up. Glenoid version and inclination, overall and by glenoid component type (PE and TM).

	Overall	PE	TM
	*n* = 91	*n* = 47	*n* = 44
**Pre-operative** Walch classification(missing *n* = 1)	A1 = 29; A2 = 6; B1 = 19; B2 = 30; B3 = 1; C = 5; D = 0 (*n* = 1)	A1 = 18; A2 = 2; B1 = 12; B2 = 11; B3 = 1; C = 3; D = 0	A1 = 11; A2 = 4; B1 = 7; B2 = 19; B3 = 0; C = 2; D = 0 (*n* = 1)
**Post-op 6 weeks** Lazarus score ^	0 = 90; 1 = 1	0 = 46; 1 = 1	0 = 44
**Post-op 2 years** Lazarus score ^	0 = 70; 1 = 15; 2 = 6	0 = 34; 1 = 9; 2 = 4	0 = 36; 1 = 6; 2 = 2
**Version** [°], mean ± SD			
**Pre-operative**	−19.41 ± 8.61	−18.94 ± 8.76	−19.93 ± 8.52
**Post-op 6 weeks**	−17.72 ± 8.53	−16.92 ± 8.39	−18.59 ± 8.69
Difference (pre-6wk)	1.86 ± 8.66	2.09 ± 9.09	1.60 ± 8.28
p-value	0.045 *	<0.0001 **	0.211
**Post-op 2 years**	−18.24 ± 9.00	−17.76 ± 7.87	−18.73 ± 10.10
Difference (6wk-2Y)	1.48 ± 8.88	1.38 ± 8.86	1.60 ± 9.01
p-value	0.119	0.303	0.249
**Inclination** [°], mean ± SD			
**Pre-operative**	11.48 ± 7.07	11.26 ± 6.81	11.72 ± 7.42
**Post-op 6 weeks**	5.87 ± 6.31	6.08 ± 4.94	5.64 ± 7.58
Difference (pre-6wk)	−5.36 ± 7.33	−5.11 ± 6.97	−5.63 ± 7.77
*p*-value	<0.0001 **	<0.0001 **	<0.0001 **
**Post-op 2 Year**	6.38 ± 6.33	6.42 ± 5.29	6.34 ± 7.30
Difference (6wk-2Y)	−5.07 ± 7.40	−5.16 ± 7.62	−4.98 ± 7.25
p-value	<0.0001 **	<0.0001 **	<0.0001 **

*p*-value (* *p* < 0.05; ** *p* < 0.01). ^ Lazarus score 0–5 [16] for PE glenoids and adapted Lazarus score for TM glenoids (0 = no loosening; 1 = loosening around superior or inferior part of component; 2 = loosening around superior and inferior part of component; 3 = loosening around superior or inferior and partial posterior part of component; 4 = loosening near complete around entire component; 5 = gross loosening around entire component). PE = Poly-Ethylene; TM = Trabecular Metal; SI = Superior–Inferior; AP = Anterior–Posterior.

**Table 2:** Glenoid component offset superior–inferior (SI) and anterior–posterior (AP), and humeral head centering measurements SI and AP at six weeks and two years postoperative.

	6 Weeks Postoperative	2 Years Postoperative	*p*-Value
**Glenoid component offset SI [mm]**			
**Overall**, mean ± SD	−0.32 ± 1.80	−0.13 ± 1.68	0.1685
**PE-glenoid**, mean ± SD	−0.38 ± 1.60	−0.28 ± 1.77	0.4066
**TM-glenoid**, mean ± SD	−0.25 ± 2.00	0.02 ± 1.60	0.2712
**Glenoid component offset AP [mm]**			
**Overall**, mean ± SD	1.91 ± 2.65	1.18 ± 2.07	0.0164 *
**PE-glenoid**, mean ± SD	2.15 ± 2.72	1.40 ± 2.20	0.109
TM-glenoid, mean ± SD	1.66 ± 2.58	0.95 ± 1.92	0.0738
**Humeral head centering SI [mm]**			
**Overall**, mean ± SD	0.68 ± 2.00	1.38 ± 3.03	0.0140 *
**PE-glenoid**, mean ± SD	0.66 ± 1.47	1.45 ± 3.28	0.0982
**TM-glenoid**, mean ± SD	0.69 ± 2.47	1.31 ± 2.77	0.0517
**Humeral head centering AP [mm]**			
**Overall**, mean ± SD	1.06 ± 2.31	1.52 ± 2.91	0.2564
**PE-glenoid**, mean ± SD	1.22 ± 2.40	1.98 ± 3.46	0.3113
**TM-glenoid**, mean ± SD	0.89 ± 2.23	1.05 ± 2.16	0.6194

*p*-value (* *p* < 0.05).

**Table 3:** Inter-rater reliability of radiographic evaluations assessed via intraclass correlation coefficients (ICC).

	Pre-Operative	Post-Op 6 Weeks	Post-Op 2 Years
**Glenoid version**	0.797	0.785	0.838
**Glenoid inclination**	0.818	0.709	0.812
**Glenoid component offset SI**	-	0.541	0.61
**Glenoid component offset AP**	-	0.68	0.565
**Humeral head centering SI**	-	0.565	0.71
**Humeral head centering AP**	-	0.277	0.636

**ICC** values less than 0.5 are indicative of poor reliability, values between 0.5 and 0.75 indicate moderate reliability, values between 0.75 and 0.9 indicate **good** reliability, and values greater than 0.90 indicate **excellent** reliability.

**Table 4:** Logistic regression models for glenoid component position and glenoid radiolucency.

Glenoid Component Radiolucency	Odds Ratio	*p*-Value
**Version at 6 wk**	1.026	0.6249
**Version (delta from pre-op)**	0.960	0.3698
**Pre-op Walch**	1.129	0.6349
**Female**	4.851	0.0088 *
**Age**	0.929	0.0940
**BMI**	0.952	0.2703
**Dominant side—Operative side**	0.411	0.1261
**Inclination at 6wk**	1.032	0.5537
**Inclination (delta from pre-op)**	0.968	0.4402
**Pre-op Walch**	1.009	0.9616
**Female**	5.007	0.0074 *
**Age**	0.928	0.0970
**BMI**	0.945	0.1933
**Dominant side—Operative side**	0.378	0.1084
**Humeral head centering (AP) at 6wk**	1.123	0.3522
**Humeral head centering (SI) at 6wk**	0.897	0.3754
**Female**	4.432	0.0094 *
**Age**	0.947	0.1915
**BMI**	0.942	0.1790
**Dominant side—Operative side**	0.415	0.1299

Glenoid component radiolucency based on two-year radiographic assessment. * *p* < 0.05.

**Table 5:** Linear regression models for two-year PROMS.

	*p*-Value
**ASES**	
**Glenoid component radiolucency at 2Y**	0.4818
**Female**	0.1357
**Age**	0.6489
**BMI**	0.4249
**Dominant side—Operative side**	0.6204
**WOOS**	
**Glenoid component radiolucency at 2Y**	0.3936
**Female**	0.1727
**Age**	0.2537
**BMI**	0.9713
**Dominant side—Operative side**	0.7051
**EQ5D slider**	
**Glenoid component radiolucency at 2Y**	0.7277
**Female**	0.5313
**Age**	0.2771
**BMI**	0.3746
**Dominant side—Operative side**	0.5998

Glenoid radiolucency based on two-year radiographic assessment.

## Data Availability

All data generated or analyzed during this study are included in this published article. Individual data are not publicly available due to data containing information that could compromise research participant privacy/consent.
